# Delivery of Online Adaptive MRI-Guided Radiation Therapy for a Deaf Patient

**DOI:** 10.7759/cureus.27558

**Published:** 2022-08-01

**Authors:** Lauren C Linkowski, Austin J Sim, Gage Redler, Andrew S Brohl, Stephen A Rosenberg, Evan J Wuthrick

**Affiliations:** 1 Radiation Oncology, University of South Florida Morsani College of Medicine, Tampa, USA; 2 Department of Radiation Oncology, James Cancer Hospital and Solove Research Institute, The Ohio State University Wexner Medical Center, Columbus, USA; 3 Department of Radiation Oncology, H. Lee Moffitt Cancer Center & Research Institute, Tampa, USA; 4 Sarcoma Department, H. Lee Moffitt Cancer Center & Research Institute, Tampa, USA

**Keywords:** mr linac, american sign language, mr-guided stereotactic body radiotherapy, culturally competent care, magnetic resonance imaging-guided radiation therapy (mrgrt)

## Abstract

MRI-guided radiation therapy (MRgRT) enables real-time imaging during treatment and daily online adaptive planning. It is particularly useful for areas of treatment that have been previously excluded or restricted from ablative doses due to potential damage to adjacent normal tissue. In certain cases, ablative doses to metastatic lesions may be justified and treated with MRgRT using video-assisted gated breath-hold adjustments throughout delivery. The workflow relies on patient biofeedback and auditory cues.

A 74-year-old deaf male with a history of prostate cancer status post prostatectomy was found to have an enlarged cervical lymph node, which was excised with histopathology demonstrating Merkel cell carcinoma. Approximately one year after treatment with two cycles of pembrolizumab, which was subsequently discontinued due to toxicity, surveillance imaging demonstrated an enlarging left adrenal nodule. It was initially stable for an additional seven months with pembrolizumab rechallenge but was again found enlarged on subsequent imaging. The patient underwent MRg stereotactic body radiation therapy (MRgSBRT) to a total dose of 60 Gy in five fractions to this isolated site of progression. The patient was equipped with mirrored glasses to view the tracking structure with respect to gating the boundary structure, and the traditional reliance on verbal cues for coaching was reimagined to rely on visual cues instead. Follow-up positron emission tomography/CT (PET/CT) two weeks after treatment demonstrated interval resolution of the left adrenal metastatic nodule and a return to symmetric bilateral adrenal gland metabolic activity. The necessary MRgSBRT treatment for single metastatic lesions near normal tissue structures relies on verbal cues and coaching. However, deaf patients are unable to receive this treatment according to the traditional workflow model. Unique opportunities exist for the implementation of culturally competent care for the Deaf community, relying more heavily on visual cues, in radiation oncology practice.

## Introduction

MRI-guided radiation therapy (MRgRT) allows for real-time imaging during treatment and daily online adaptive planning, especially in areas where treatment may be restricted to account for normal tissue dose and organ motion [1]. The ability to apply these benefits allows for the delivery of previously unattainable ablative doses of radiation via MRg stereotactic body radiation therapy (MRgSBRT) in multiple disease sites for both primary and metastatic lesions [2,3]. As seen in multiple phase II clinical trials, catering to a subset of patients with limited metastases may redefine our definition of “metastatic disease” and potentially justify delivering definitive ablative doses to a limited number of metastatic sites [4-6]. The use of real-time image guidance during treatment has required a change in workflow for adaptive MRgSBRT, using video-assisted gated breath-hold and adjustments throughout delivery, as well as auditory reassurance and assistance [7]. The workflow involves a deep inspiratory breath-hold (DIBH) accompanied by patient biofeedback displayed on a screen to track the structure's position with respect to a gating boundary structure. Currently, no guidelines exist for treating deaf patients with cancer. This includes a lack of instruction for radiation oncologists for treating deaf patients [8]. Given the traditional reliance on verbal cues for breath-holds during MRgSBRT delivery, the treatment of deaf patients presents a unique challenge.

## Case presentation

A 74-year-old man with a remote history of localized prostate cancer status post prostatectomy developed painless, enlarged cervical lymph nodes. Excision of an involved left cervical node revealed a 2 x 1.5 x 4-cm mass with pathologic features consistent with Merkel cell carcinoma of unknown primary. A follow-up CT scan showed disease in the left neck at levels I and II but revealed no other areas of metastatic disease. He then received two cycles of pembrolizumab and achieved a rapid and complete clinical and radiographic response. Further therapy, however, was discontinued due to toxicity, and the patient opted for a transition to close surveillance.

He was disease-free for approximately one year, but surveillance imaging revealed an enlarging left adrenal nodule. He was rechallenged with pembrolizumab. On pembrolizumab rechallenge, he achieved a partial response to therapy initially, but a positron emission tomography/CT (PET/CT) at seven months demonstrated the regrowth of the left adrenal mass to 2.7 x 3.3 cm (previously 1.2 x 1.0 cm).

Given his isolated site of disease progression, he was offered options for definitive local therapy, including adrenalectomy or stereotactic body radiation therapy (SBRT). Given his age, use of immunotherapy, and comorbid medical conditions (arthritis, hyperlipidemia, hypertension, and lymphadenopathy), he opted for SBRT with continued concurrent pembrolizumab.

Imaging suggested that the proximity of a loop of bowel would prevent ablative doses to significant portions of the lesion with conventional SBRT, requiring adaptive MRgSBRT. He thus underwent MR simulation in the supine position with his left arm above his head without further immobilization on the MR-linac using the DIBH technique. A CT simulation was then completed in the same position using DIBH; images were fused prior to contouring to provide electron density data. After the gross tumor volume (GTV) was delineated on the planning MRI, a 3-mm expansion was used to create the low-dose planning target volume (PTV_4000). The bowel, duodenum, and stomach were then contoured and expanded by 10 mm (BDS+10). A high-dose planning target volume (PTV_6000) was then created by subtracting BDS+10 from PTV_4000. This PTV was adjusted based on the BDS recontoured at the time of each treatment for daily plan adaptation. A conformal 6X photon radiation plan was designed using a simultaneous integrated boost (SIB) approach via step-and-shoot fixed-field IMRT (Table 1). Given the ability to adapt the patient's plan on a daily basis, he was not treated NPO.

**Table 1:** Summary of initial plan metrics PTV: planning target volume; Vxx Gy: volume (cm^3^ or %) receiving xx Gy

Structure	Maximum dose (Gy)	Minimum dose (Gy)	Volumetric dose	
PTV_6000	77.36	55	V60Gy = 99.76%	
PTV_4000	77.36	38	V40Gy = 99.56%	
Spinal cord	10.45			
Bowel	38		V35Gy = 0.49 cc	V32Gy = 1.76 cc
Stomach	21.99		V35Gy = 0 cc	V32Gy = 0 cc
Duodenum	38		V35Gy = 0.14 cc	V32Gy = 0.53 cc
Kidney_L			Mean = 7.86 Gy	
Kidney_R			Mean = 2.23 Gy	

**Figure 1:**
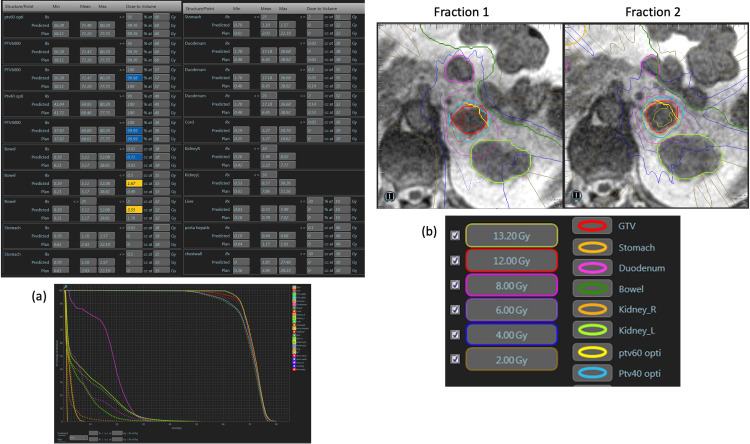
Representative adaptive fractions (a) Prediction of dose from the original plan on the anatomy of the day versus the new adaptive plan. The dose-volume histogram (DVH) of the new plan (dotted lines) show near identical coverage of the target volumes of the original plan (solid lines). (b) Isodose lines from fractions 1 and 2 as delivered after adaptation

**Figure 2:**
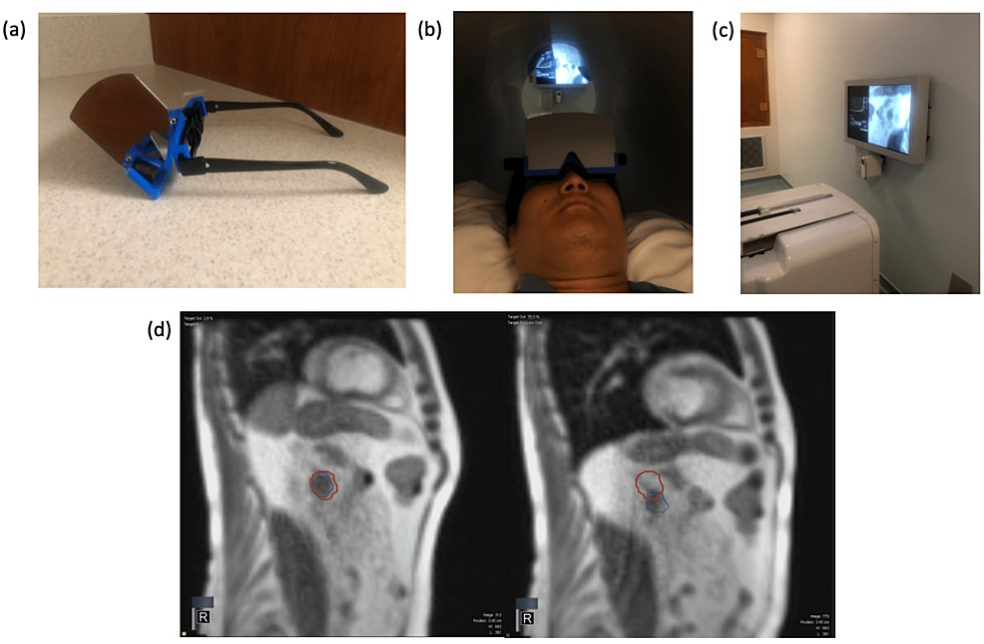
Magnetic resonance (MR) fraction delivery setup (a) Mirrored glasses used by the patient while lying in the bore of the MR-linac (b), allowing the patient to see the screen (c) with the tracking structure and boundary structure (d) target tracking structure on sagittal T1 MRI

A total of 60 Gy in five fractions was delivered on non-consecutive days using daily MR guidance and adaptation with respiratory gating. Four out of the five fractions required online adaptive replanning prior to fraction delivery due to unacceptable doses to organs at risk (OAR) (Figure 1). For typical fractions of MRgSBRT, we equip patients with mirrored glasses to view real-time imaging of a tracking structure with respect to a boundary structure (Figure 2).

Therapists provide verbal coaching in conjunction with this visual feedback to coordinate breath-holds with treatment delivery and free breathing with gantry movement during step-and-shoot IMRT delivery. However, we were unable to provide verbal coaching for this patient. During CT simulation and with the assistance of an American Sign Language (ASL) interpreter, we were able to develop a plan to communicate by turning the lights in the vault on and off to signal him to hold his breath and breathe. The ASL interpreter, as a vital member of the patient’s care team, was also present during treatment and provided traditional, visual communication between the care team and the patient. The patient was instructed to breathe normally when the lights were on. He was instructed to perform DIBH when the lights were off. In addition to the room lights, the patient was able to visually track his tumor with the assistance of the mirrored glasses. He was aware that the lights would remain off while the tracking structure was within the gating structure, indicating that he should remain in DIBH. The patient received a scheduled infusion of pembrolizumab between the fourth and fifth fraction of MRgSBT and tolerated this treatment well with no acute toxicities. He was subsequently continued on 200 mg pembrolizumab intravenously every three weeks. Follow-up PET/CT completed two weeks after MRgSBRT demonstrated interval complete resolution of the left adrenal metastatic nodule and a return to symmetric bilateral adrenal gland metabolic activity. At a two-month follow-up, he noted non-specific abdominal cramping and bloating. Laboratory studies, including a complete blood count and comprehensive metabolic panel, were unremarkable. At eight-month and 18-month follow-ups, he had isolated sites of progression to the right femoral head and subcarinal lymph node, respectively, both of which were treated with radiation therapy.

## Discussion

In this case, adaptive MRgSBRT was critical for the successful treatment of this patient’s adrenal lesion with minimal damage to adjacent normal tissue structures. The treatment modality is associated with higher accuracy and greater coverage in adrenal tumors [2]. The ability to reoptimize treatment plans based on the daily movement of at-risk organs leads to significant improvement in target coverage, at-risk organ sparing, and dose escalation [9,10]. SBRT has previously been reported to be efficacious to treat Merkel cell carcinoma, either alone [11], or in conjunction with immunotherapy [12]. Compared to standard SBRT, MRgSBRT treatments are longer, and effective coaching is imperative to minimize on-table and treatment times [3].

Treating a deaf patient, who uses an ASL interpreter for communication, presented a challenge in terms of delivering the most effective treatment. ASL is a complete language that uses its own grammar and syntax to communicate effectively with members of the Deaf community [13]. Deaf patients are at a disadvantage when it comes to the shared decision-making process and communication with English-speaking providers. Participation in the shared decision-making process relies on both accessible communication and the cultural competency of providers who treat deaf patients [14]. In our case, the ASL interpreter was an integral member of the care team, and ultimately the key participant in treating the patient. Primary care settings have attempted to implement more appropriate care for deaf patients, which has served to increase culturally and linguistically competent providers; however, these changes have yet to be explored among oncology practitioners, including radiation oncologists [8]. Hearing loss directly affects 23% of Americans aged 12 years or older, and 2.5% are classified as having severe to profound hearing loss - 75% of whom are over the age of 60 years [15]. The Americans with Disabilities Act, last updated in 2010, states that healthcare providers must ensure that communication with patients with hearing, vision, and speech disabilities is as effective as communication with other patients [16,17]. Effective communication includes qualified interpreters provided by the healthcare facility, written materials, open and closed captioning, and other visual-based accommodations [17].

## Conclusions

In our case, this novel treatment, and the voice cues by which it was delivered did not meet the standard for effective communication with the patient. We utilized the entire team, including the patient and ASL interpreter, to develop a solution that allowed everyone to feel comfortable with the treatment delivery. At this time, our team is unaware of any on-screen text option on our treatment machine to assist in treating deaf or hard-of-hearing patients. These statistics provide the tools for a necessary conversation about accommodating patients with significant hearing loss when using novel radiotherapy technology. Steps must be taken towards physician education focusing on the place of individualized care to accommodate the diversity of the Deaf community, as well as accessibility to certified ASL interpreters and resources of varying linguistic approaches. Opportunities that allow physicians to gain exposure to both culturally competent care and basic proficiency in ASL throughout training should be considered moving forward. Oncology practitioners, including radiation oncologists, should set a standard for caring for deaf patients with guidelines that can be applied to all specialties of oncological care. We have created a practical innovation, with an already involved workflow, to provide culturally competent care and offer the most appropriate technology.
